# Alteration in Fetal Cardiac Function at Mid-Gestation Among Pregnancies Subsequently Complicated by Preeclampsia, Fetal Growth Restriction and Gestational Diabetes Mellitus: A Literature Review

**DOI:** 10.3390/jcm15051845

**Published:** 2026-02-28

**Authors:** Iulia Huluță, Livia-Mihaela Apostol, Nicoleta Gana, Radu Botezatu, Anca-Maria Panaitescu

**Affiliations:** 1 Faculty of Medicine, Carol Davila University of Medicine and Pharmacy, 020021 Bucharest, Romania; iuliahuluta16@gmail.com (I.H.);; 2 Clinical Hospital of Obstetrics and Gynaecology Filantropia, 011171 Bucharest, Romania

**Keywords:** fetal speckle-tracking echocardiography, mid-gestation cardiac function, preeclampsia, gestational diabetes mellitus, fetal growth restriction

## Abstract

Preeclampsia (PE), fetal growth restriction (FGR), and gestational diabetes mellitus (GDM) complicate approximately 15–20% of pregnancies and represent major contributors to perinatal morbidity, mortality, and long-term cardiovascular risk in offspring. Increasing evidence from longitudinal cohort studies indicates that adult cardiovascular disease, including hypertension, coronary artery disease, and stroke, may be programmed in utero through early alterations in fetal cardiac structure and function. Two-dimensional speckle-tracking echocardiography (2D-STE) has emerged as the most sensitive non-invasive technique for detecting subclinical myocardial deformation, often preceding abnormalities detected by conventional Doppler or biometric parameters. While numerous third-trimester studies have demonstrated impaired global longitudinal strain (GLS), altered ventricular geometry, and diastolic dysfunction in established disease, data from mid-gestation (18–28 weeks), the critical preclinical window, remain extremely limited. Therefore, this review aims to systematically synthesize the available evidence on fetal cardiac deformation parameters assessed by 2D-STE at mid-gestation in pregnancies that subsequently developed PE, FGR, or GDM, in order to identify the earliest detectable signatures of fetal cardiovascular programming and highlight key knowledge gaps that must be addressed prior to clinical implementation.

## 1. Introduction

Maternal and fetal disorders such as preeclampsia, fetal growth restriction, and gestational diabetes mellitus are major contributors to adverse perinatal outcomes and long-term cardiovascular morbidity in both mother and offspring [1,2,3,4,5,6,7]. Increasing evidence suggests that the fetal heart undergoes early functional and structural adaptations in response to the altered intrauterine environment preceding the clinical onset of these complications [8,9,10,11,12].

2D-STE has emerged as a valuable technique for quantifying fetal myocardial deformation, offering a sensitive assessment of subclinical ventricular dysfunction before conventional Doppler or biometry parameters become abnormal [13,14,15]. Global longitudinal strain (GLS), strain rate, and other STE-derived indices provide insight into early myocardial mechanics and placental–cardiac interactions, reflecting subtle shifts in preload, afterload, and myocardial compliance [14,15,16].

Exposure to PE, FGR or GDM profoundly influences neonatal and long-term cardiovascular health through mechanisms of fetal programming.

Offspring exposed to hypertensive disorders of pregnancy (HDP) have modestly higher blood pressure later in life (pooled mean difference approximately +2.4 mmHg systolic and +1.4 mmHg diastolic across childhood to adulthood), although estimates vary by study design and adjustment strategy [17]. In a large Nordic population-based cohort of approximately 8.5 million births, prenatal exposure to maternal preeclampsia was independently associated with a 33% higher risk of ischemic heart disease and a 34% higher risk of stroke in offspring during childhood and young adulthood, with effect estimates persisting after adjustment for preterm birth and small for gestational age status and being more pronounced for severe preeclampsia phenotypes [18].

FGR infants are born with significant cardiac remodelling: a 2024 meta-analysis of 1247 neonates reported increased left-ventricular mass index (+1.5 g/m^2^), reduced right-ventricular fractional area change (−5.2%), and persistent arterial stiffness up to age 5 years (carotid intima-media thickness + 0.04 mm) [19,20].

Fetuses of pregnancies complicated by GDM demonstrate altered cardiac geometry and subclinical biventricular systolic dysfunction in late gestation, characterized by more globular ventricles and reduced global longitudinal strain (RV GLS −16.4% vs. −18.5% and LV GLS −20.1% vs. −21.3% in controls). These functional alterations persist into infancy, with evidence of impaired systolic and diastolic performance after multivariable adjustment [2]. Population-based cohort data indicate that offspring exposed to maternal diabetes during pregnancy exhibit a modestly increased risk of early-onset cardiovascular disease (approximately 20–30%), with higher estimates reported in pregestational diabetes and in the presence of maternal cardiovascular comorbidities. However, evidence specific to isolated GDM remains limited and is influenced by historical diagnostic criteria and relatively young follow-up populations [5]. These neonatal cardiac signatures, hypertrophy, impaired myocardial deformation, and haemodynamic instability, represent the earliest clinical manifestations of intrauterine programming and underscore the urgent need to identify prenatal precursors. Mid-gestation 2D-STE offers a unique window to detect the inception of this process before irreversible structural changes occur.

Building on prior research that highlighted early deviations in fetal myocardial performance at mid-gestation, this review aims to synthesize current evidence on speckle-tracking-derived cardiac function in pregnancies that subsequently developed PE, FGR or GDM. We aim to delineate consistent trends in functional adaptation, evaluate methodological consistency, and identify knowledge gaps that could guide future prospective and mechanistic studies. Mid-gestation, typically between 18 and 28 weeks, represents a pivotal period for evaluating these adaptive changes, as it precedes the overt manifestation of maternal disease and fetal compromise.

Many studies published over the past two decades have demonstrated varying patterns of reduced ventricular GLS, altered strain rate, and ventricular asymmetry in fetuses from pregnancies complicated by PE, FGR or GDM, but in late gestation.

## 2. Materials and Methods

### 2.1. Protocol and Search Strategy

This literature review, without meta-analysis, was conducted in accordance with the PRISMA 2020 guidelines. A comprehensive search of PubMed, Scopus, and Embase was performed to identify studies published between January 2000 and November 2025 that evaluated fetal cardiac function at mid-gestation using 2D-STE in pregnancies that later developed preeclampsia, FGR or GDM. The full electronic search strategies for all databases, including exact Boolean operators and query syntax, are provided in Supplementary Table S1 in accordance with PRISMA reporting recommendations. The review protocol was not prospectively registered; however, the eligibility criteria, outcomes, and analysis plan were defined a priori and are fully reported in the Methods.

The search strategy combined controlled vocabulary and free-text terms related to fetal echocardiography, speckle tracking, strain, myocardial deformation, preeclampsia, growth restriction, and GDM. Additional studies were identified by screening the reference lists of relevant articles and author-known sources. Searches were limited to human studies published in English involving in utero STE assessment of fetal cardiac function. Both prospective and retrospective observational designs (cohort, case–control, cross-sectional) were eligible; no randomized controlled trials were identified. Studies that performed STE at mid-gestation (approximately 18–28 weeks) were prioritized, though later gestational assessments were reviewed when relevant for longitudinal interpretation or discussions.

### 2.2. Study Selection

**Figure 1:**
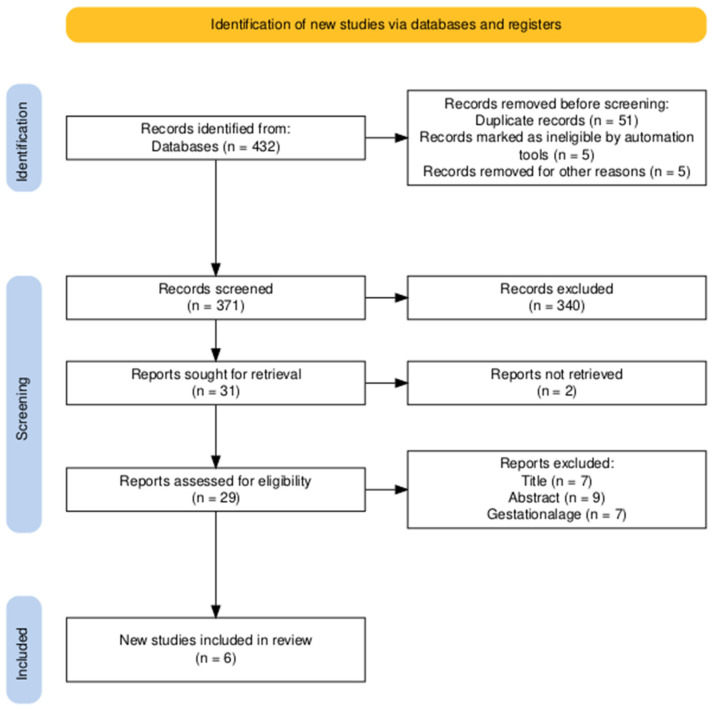
Prisma Flow diagram.

After duplicate removal, titles and abstracts were screened for relevance by two independent reviewers, followed by full-text evaluation of potentially eligible articles. Discrepancies were resolved by consensus. The PRISMA flow diagram (Figure 1) summarizes the selection process.

In total, 6 studies met the inclusion criteria for qualitative synthesis, after initial identification of >400 records and full-text review of 29 manuscripts.

### 2.3. Data Extraction

For each included study, data were extracted regarding authorship, publication year, country, study design, sample size (complicated and control groups), gestational age at cardiac assessment, type of pregnancy complication, and STE parameters evaluated, especially left and right ventricular global longitudinal strain, strain rate, and indices of systolic and diastolic function. Key findings describing how each complication affected fetal cardiac performance were summarized qualitatively.

### 2.4. Quality Assessment

The methodological quality of cohort and case–control studies was assessed using the Newcastle–Ottawa Scale (NOS) adapted for fetal observational research, which evaluates selection, comparability, and outcome domains (score range 0–9). Overall, study quality was moderate to good. Most investigations were prospective but cross-sectional (single-time-point) studies with group sizes of approximately 30–100 fetuses. Common limitations included heterogeneity in STE analysis protocols, lack of examiner blinding, and incomplete adjustment for confounders such as fetal size or maternal characteristics. Most studies achieved NOS scores between 5 and 8, indicating satisfactory methodological rigour but some risk of bias in comparability.

### 2.5. Data Synthesis

Due to substantial heterogeneity across included studies in terms of study design, STE vendor software platforms, image acquisition protocols, frame rate settings, myocardial segmentation approaches, reproducibility reporting, gestational age at assessment, and outcome definitions, a quantitative meta-analysis was not feasible. Additional variability related to feasibility and image quality constraints at mid-gestation (18–28 weeks), including fetal motion, suboptimal acoustic windows, and operator dependency, further limited direct comparability between studies. Therefore, findings were synthesized qualitatively and organized according to pregnancy complications.

## 3. Results

A total of 6 studies, published between 2009 and 2025, met the inclusion criteria and were included in the qualitative synthesis. All were observational in design, either prospective cohort or cross-sectional analyses; no randomized controlled trials were identified. The PRISMA flow diagram (Figure 1) summarizes the study selection process. Most included papers were published within the last decade, reflecting the progressive introduction and validation of fetal 2D-STE in clinical and research settings. Across all studies, STE was performed predominantly at mid-gestation (18–28 weeks), corresponding to the period of detailed fetal anatomic evaluation, with several studies including additional assessments in the third trimester for comparison (Table 1).

Control groups consisted of fetuses from uncomplicated singleton pregnancies. The primary outcomes evaluated were indices of systolic myocardial function, such as global longitudinal strain, strain rate, and fractional area change. Several studies also assessed diastolic performance (e.g., E/A ratio, myocardial performance index) and cardiac geometry parameters (sphericity index, wall thickness, chamber dimensions).

### 3.1. Preeclampsia and Fetal Cardiac Function at Mid-Gestation

Mid-gestation assessments of fetal cardiac function in pregnancies that later develop PE reveal very subtle early alterations, though the magnitude of change is minimal (Table 1).

In the largest prospective cohort to date (*n* = 5801 singleton pregnancies scanned at 19 + 0–23 + 6 weeks), fetuses from pregnancies that subsequently developed PE (*n* = 179, 3.1%) exhibited subtle but statistically significant LV systolic impairment compared with uncomplicated pregnancies (*n* = 5608) [8]. Unadjusted mean LV GLS was less negative in the future-PE group (−22.2% [95% CI −23.3 to −21.1] vs. −23.3% [−24.7 to −21.9]; *p* = 0.002), corresponding to a multivariable-adjusted mean difference of +0.8% (95% CI +0.3 to +1.3%; *p* = 0.002) after correction for gestational age, fetal biometry, maternal age, BMI, ethnicity, and other confounders. LV ejection fraction was also mildly reduced (unadjusted 69.2% [66.7–71.8] vs. 72.3% [71.9–72.8]; adjusted difference −1.2%, 95% CI −2.1 to −0.3%; *p* = 0.008). Right-ventricular GLS (−21.1% [−21.7 to −20.4] vs. −20.5% [−20.7 to −20.4]; *p* = 0.12), sphericity indices, cardiothoracic ratio, and all other morphometric parameters remained comparable between groups. No correlations were observed between fetal cardiac indices and second-trimester uterine artery pulsatility index or placental growth factor multiples of the median. The authors concluded that these extremely small absolute differences (≈1–2%) represent subclinical LV functional deviation; however, their clinical significance at mid-gestation remains uncertain [8].

Evidence indicates that mid-gestation PE is associated only with minor, statistically detectable but clinically negligible changes in fetal myocardial performance. Detecting such small differences requires large sample sizes and high-sensitivity techniques such as 2D-STE, as smaller studies have not consistently replicated these findings.

### 3.2. Gestational Diabetes Mellitus and Fetal Cardiac Function

As shown in Table 1, in pregnancies subsequently complicated by GDM, mid-gestation fetal myocardial deformation assessed by 2D-STE is preserved when evaluated before clinical diagnosis, whereas functional impairment becomes evident after maternal hyperglycemia is established [11,12,21].

The only large pre-diagnostic study (Huluta et al., 2023; *n* = 5620 singleton pregnancies scanned at 19–23 weeks) identified 470 women who later developed GDM [11]. After adjustment for gestational age, maternal characteristics, and fetal biometry, no differences were observed in left-ventricular (LV) global longitudinal strain (GLS) (adjusted δ = 0.3%, 95% CI −0.4 to +1.0%) or right-ventricular (RV) GLS (adjusted δ = 0.22%, 95% CI−0.24 to +0.68%) between fetuses of future-GDM and normoglycaemic pregnancies. Minor morphological differences were limited to mildly increased interventricular septal thickness (adjusted δ = 0.04 mm, 95% CI 0.03–0.05 mm) and larger left atrial area (adjusted δ = 0.04 cm^2^, 95% CI 0.04–0.05 cm^2^), with LV ejection fraction marginally higher in the future-GDM group (adjusted δ = +0.02%, *p* < 0.05) [11].

In contrast, longitudinal studies performed after GDM diagnosis demonstrated clear systolic impairment from the early third trimester onward. Yovera et al. (2021; *n* = 336, scans at/after diagnosis, second-trimester subset 24–32 weeks) reported preserved LV GLS (adjusted mean difference +0.2%, 95% CI −0.1 to +0.5%, non-significant) but significantly reduced RV GLS (adjusted mean difference +0.7%, 95% CI +0.3 to +1.1%, *p* < 0.05) and lower RV tricuspid annular plane systolic excursion (TAPSE) in GDM fetuses [12]. Wang et al. (2021; *n* = 116, scans after diagnosis, 24–40 weeks) observed marked biventricular systolic dysfunction with LV GLS of −18.26 ± 1.39% versus −22.70 ± 1.34% in controls and RV GLS of −18.52 ± 1.35% versus −22.74 ± 1.34% in controls, accompanied by reduced fractional area change in both ventricles and a rounder cardiac shape (lower global sphericity index 1.21 vs. 1.27, *p* = 0.000) [21].

Collectively, these findings indicate that no measurable differences in fetal myocardial deformation were detected prior to clinical diagnosis of GDM and that functional changes emerge only after sustained exposure to diabetes mellitus.

### 3.3. Fetal Growth Restriction and Fetal Cardiac Function

FGR encompasses a spectrum ranging from constitutionally small-for-gestational-age (SGA) fetuses to severe early-onset pathological growth restriction secondary to placental insufficiency [20]. Mid-gestation 2D-STE findings vary according to FGR severity and timing of onset.

In severe early-onset FGR, case series evidence indicates pronounced biventricular systolic impairment detectable as early as 20–22 weeks, well before overt Doppler abnormalities. Neacșu et al. reported two cases of severe placental FGR scanned at 20–22 weeks using TomTec arena 2020 software, demonstrating markedly reduced left-ventricular global longitudinal strain (LV-GLS) of −15.2% and −14.8% (vs expected mid-gestation norms ≈ −20 to −22%) and right-ventricular GLS (RV-GLS) of −16.5% and −15.9% (vs norms ≈ −21 to −24%), accompanied by reduced cardiac output and increased intraventricular dyssynchrony (>45 ms) [22].

In contrast, in fetuses destined to develop late-onset SGA/FGR, longitudinal STE reveals early isolated right-ventricular dysfunction that precedes any growth or Doppler abnormality. van Oostrum et al. prospectively followed 136 fetuses (including 12 who later became FGR) from 18 weeks using TomTec software [19]. At 18–21 weeks, RV-GLS was already significantly less negative in the future-SGA group (−21.4 ± 3.8% vs. −24.9 ± 3.1% in controls; *p* = 0.001), with the difference persisting at 22–25 weeks (−22.1 ± 3.5% vs. −25.6 ± 3.0%; *p* < 0.001). LV-GLS remained comparable between groups until after 26 weeks, confirming that subclinical RV systolic dysfunction represents the earliest detectable functional marker in late-onset pathological growth restriction [19].

These findings suggest a severity-dependent pattern: severe early-onset FGR exhibits biventricular GLS reduction and dyssynchrony at 20–22 weeks, whereas late-onset SGA/FGR is characterized by isolated RV impairment detectable from as early as 18–21 weeks.

## 4. Discussion

The most clinically relevant finding of this literature review is that mid-gestation (18–28 weeks) may represent a phase of relative myocardial preservation in pregnancies destined to develop PE, GDM, or FGR, despite the presence of placental or metabolic stress that is already detectable by angiogenic biomarkers or uterine artery Doppler.

**Figure 2:**
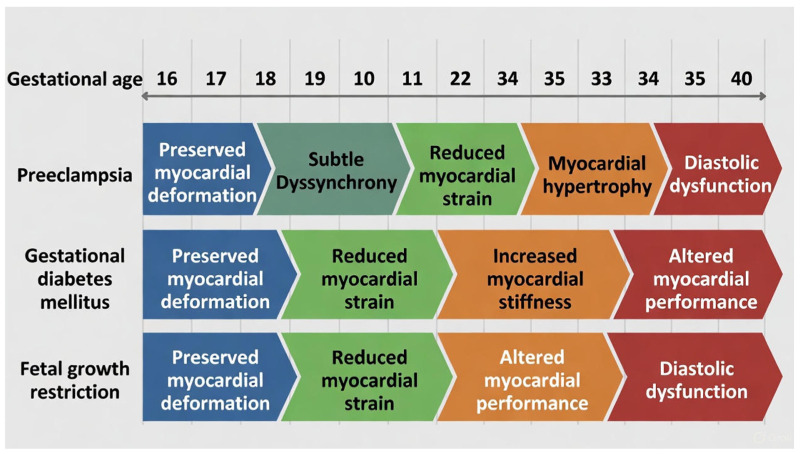
Temporal evolution of fetal myocardial adaptation in pregnancies subsequently complicated by PE, GDM, or FGR, as detected by STE. Horizontal axis: gestational age (weeks). Each row represents one complication; colour-coded arrows indicate progression of functional/structural changes based on current evidence. Blue = preserved function; green = early subclinical change; orange = established remodelling; red = overt dysfunction. The temporal sequence illustrated is conceptual and derived from heterogeneous observational datasets.

Mid-gestation assessment in pregnancies that later develop PE reveals only subtle fetal cardiac alterations, supporting the concept that functional impairment develops secondary to placental disease rather than preceding it. The fetal heart exhibits only trivial left-ventricular hypercontractility or mild strain reduction. These changes are statistically significant in very large cohorts but are far below thresholds of clinical relevance (Figure 2). This suggests that the fetal myocardium remains compensated until placental dysfunction becomes severe enough to trigger overt remodelling in the third trimester. The clinical utility of a 1% strain variation at 20 weeks has not yet been established [8]. In the large prospective cohort involving over 5800 pregnancies, fetuses at 19–24 weeks who later developed PE exhibited a slightly lower (less negative) LV GLS and marginally lower ejection fraction compared with controls. No differences were observed in right ventricular (RV) strain or dimension indices. The absolute magnitude of difference was extremely small (≈1–2%), likely below clinical relevance. These findings suggest mild early left ventricular adaptation, possibly an early compensatory hypercontractility in response to subtle placental resistance or intermittent hypoperfusion. As gestation advances and placental vascular pathology progresses, these adaptive changes may evolve into ventricular remodelling and diastolic dysfunction, as reported in late-onset PE cohorts [1]. The absence of significant correlation between fetal strain indices, uterine artery Dopplers, or placental growth factor levels further indicates that STE alone cannot serve as an early biomarker for PE, though it offers mechanistic insights into the evolving placental-cardiac axis.

At 18–28 weeks, therefore, the fetal myocardium remains functionally compensated, despite maternal vascular maladaptation. The clinical implication is that early fetal cardiac imaging cannot predict who will develop PE, but it may reveal the earliest cardiovascular signatures of placental stress. Future longitudinal studies integrating mid-gestation STE with biomarkers of placental function

Thus, fetal cardiac STE at mid-gestation should not be regarded as a stand-alone predictive tool for PE, but it provides valuable insight into early placental–cardiac coupling mechanisms. The concept of fetal cardiac “programming” in PE likely manifests later in gestation or postnatally, as shown in studies demonstrating altered cardiac structure and function in offspring of severe PE pregnancies during infancy and childhood [1,6,7,24]. Future research should examine whether prophylactic interventions, such as low-dose aspirin, could mitigate these early myocardial adaptations.

From a physiological perspective, placental disease primarily imposes increased afterload and pressure-mediated myocardial stress, whereas GDM exerts predominantly metabolic effects on fetal myocardium. In the fetal circulation, right ventricular dominance and parallel circulation architecture may explain the earlier vulnerability of RV deformation indices observed in late gestation. Mechanical dyssynchrony likely reflects early regional wall stress heterogeneity preceding global pump failure, representing a compensated adaptation phase before overt systolic dysfunction becomes detectable.

In GDM, functional indices are virtually identical to uncomplicated pregnancies throughout mid-gestation, confirming that hyperglycemia-induced myocardial changes require prolonged fetal exposure and only manifest after 28 weeks (Figure 2). This temporal pattern provides physiological reassurance for the current timing of routine anomaly scanning and underscores the importance of glycemic optimization from diagnosis onward.

Huluta et al. analyzed over 5600 fetuses that were assessed by STE at 19–23 weeks before GDM diagnosis. No significant differences in LV or RV strain, strain rate, or fractional area change were found between fetuses of mothers who later developed GDM and those who did not. Only minimal anatomical variations were noted: slightly increased interventricular septal thickness and larger left atrial area in the “future GDM” group, with a marginally higher LV ejection fraction (~2%). These differences likely reflect maternal anthropometric factors (age, BMI) rather than early metabolic impact [11].

Collectively, this evidence supports that mid-gestational fetal cardiac function remains preserved, suggesting that hyperglycemia-induced remodelling occurs later, once the fetus is exposed to sustained metabolic imbalance. By the third trimester, several independent studies demonstrate subtle biventricular dysfunction, particularly of the right ventricle. For example, Huang et al. reported at ~32 weeks that both LV and RV GLS were reduced in GDM fetuses (by 3–4% absolute), with greater impairment of RV free wall strain and preserved global geometry, indicating subclinical systolic dysfunction without overt hypertrophy [25]. Similarly, Miranda et al. (2018) observed lower diastolic strain rates and mild RV dysfunction in diabetic pregnancies (gestational and pregestational), even when conventional Doppler indices appeared normal [2,3,4,5,6,7,8,9,10,11,12,13,14,15,16,17,18,19,20,21,22,23,24,25,26,27].

These findings point to progressive fetal myocardial maladaptation in diabetic pregnancies, where chronic exposure to hyperglycemia and hyperinsulinemia promotes diastolic stiffness, altered calcium handling, and regional wall stress, particularly in the RV, which is more volume-dependent and metabolically active in utero.

From a clinical perspective, these findings suggest that mid-gestation fetal 2D STE should currently be regarded as a marker of early fetal cardiovascular adaptation, rather than a stand-alone diagnostic or prognostic tool for preeclampsia, fetal growth restriction, or gestational diabetes mellitus. Given the limited and heterogeneous evidence base, observed differences in deformation indices are typically modest and remain susceptible to platform-, software- and acquisition-related variability; therefore, 2D-STE findings at this gestational window should not, in isolation, prompt deviation from guideline-based obstetric surveillance. Nevertheless, these data are clinically informative in two respects. First, they support a biologically plausible framework whereby placental and metabolic stressors may influence fetal myocardial mechanics before overt maternal or fetal disease, highlighting a potential interval for optimization of established preventive and monitoring strategies. Second, they outline a pragmatic translational pathway in which 2D-STE may acquire clinical utility when incorporated into multimodal risk stratification together with maternal characteristics, biochemical markers and established Doppler and growth parameters, and when evaluated against clinically meaningful outcomes, including perinatal morbidity and longer-term offspring cardiovascular phenotype. At present, the most actionable implication is that pregnancies that subsequently develop PE/FGR/GDM may warrant targeted longitudinal follow-up, with standard serial fetal biometry and Doppler surveillance as the cornerstone of care, while 2D-STE is reserved for mechanistic insight and future refinement of risk prediction once methodological standardization and robust gestational age-specific reference ranges (Z-scores) are available.

Longitudinal designs measuring the same fetuses before and after GDM onset are needed to confirm the trajectory of these functional changes. In summary, GDM does not measurably alter fetal cardiac function by mid-gestation, but prolonged exposure leads to mild biventricular dysfunction, which is more pronounced in the right ventricle by late pregnancy.

FGR is associated with chronic placental underperfusion and increased fetal cardiac afterload, leading to progressive cardiovascular remodelling [23]. Available 2D-STE evidence in FGR remains limited and heterogeneous. FGR probably presents the earliest and most heterogeneous cardiac signature among placental diseases. (Figure 2) In the systematic review by van Oostrum et al., only four mid- to late-gestation studies were eligible, and global strain findings were inconsistent across cohorts, ranging from no difference vs. controls to increased GLS suggestive of compensation [24]. However, in studies that evaluated mechanical dyssynchrony, timing dispersion was consistently increased in FGR, supporting dyssynchrony as a potentially earlier marker of subclinical myocardial stress than global deformation indices.

By late gestation, worsening placental resistance and fetal hypoxemia are associated with concentric remodelling, diastolic dysfunction, and deterioration in conventional functional parameters, aligning STE changes with the broader hemodynamic continuum captured by biometry and Doppler assessment [25,26,27,28,29,30]. By term, many have evidence of impaired relaxation and early heart failure, reflected in a higher myocardial performance index (MPI) and atrial dilatation. Patey et al. showed that term FGR fetuses had concentric remodelling (smaller volumes, thicker walls) and reduced longitudinal ventricular motion compared with controls, changes that persisted into the neonatal period [31]. These findings are in line with data in small-for-gestational-age (SGA) infants, where a spectrum of cardiovascular remodelling and dysfunction across different severities of growth restriction has been described [32,33].

Importantly, STE may help distinguish constitutionally small fetuses from true placental disease: cohorts comparing late-onset FGR, SGA, and AGA fetuses suggest that abnormal Doppler-defined placental dysfunction is more likely to coincide with adverse functional signatures than isolated small size [34,35]. Overall, current data support interpreting STE as an adjunct to Doppler and clinical phenotype rather than a standalone diagnostic tool.

Mid-gestation evaluation remains particularly challenging. FGR is often diagnosed in the late second or third trimester once biometry falls below the 10th percentile and Doppler changes emerge. Some studies have instead focused on high-risk pregnancies identified by abnormal uterine artery Dopplers at 20–24 weeks, suggesting that subtle changes in cardiac shape or function may already be present in fetuses that later become growth-restricted. Neacșu et al. reported an early decline in longitudinal strain and cardiac output in fetuses that evolved into severe FGR, compared with those that remained constitutionally small but otherwise healthy [22]. These observations, though based largely on case series, support the hypothesis that compensation (preserved strain with increasing dyssynchrony) precedes decompensation (reduced strain, rising MPI and Doppler deterioration).

From a pathophysiological perspective, the progression from preserved GLS with increased dyssynchrony (compensated state) to reduced strain, elevated MPI and overt Doppler abnormalities (decompensated state) mirrors the haemodynamic continuum of placental disease. The recurring finding of increased mechanical dyssynchrony in FGR across multiple studies, including those by Krause et al. and Crispi et al., suggests that timing abnormalities may be one of the earliest and most robust markers of myocardial stress in this population [28,29]. STE thus appears most informative when interpreted alongside Doppler and biometry, rather than in isolation. Because our aim is to capture mid-gestation antecedents of later placental disease, we cite studies reporting SGA at birth as the downstream outcome when earlier phenotyping into constitutional SGA versus FGR is not yet feasible; we do not treat SGA and FGR as interchangeable diagnoses.

Clinically, if STE can reliably detect early myocardial dysfunction in FGR, it could refine surveillance and help individualize the timing of delivery. A fall in GLS or a rise in dyssynchrony could signal impending decompensation, potentially adding value beyond conventional Doppler thresholds. In summary, FGR leads to characteristic patterns of remodelling and, in more severe cases, dysfunction that STE can detect. STE holds considerable promise to refine FGR monitoring, but more robust and standardized evidence is required before it can meaningfully change clinical practice.

Although small case reports have described early abnormalities in deformation and cardiac output in severe phenotypes, these observations should be considered hypothesis-generating. Larger prospective studies with standardized acquisition/analysis protocols and longitudinal outcome correlation are required before STE parameters can be incorporated into routine FGR surveillance.

Although PE, FGR and GDM represent distinct clinical entities, they share common features of adverse intrauterine exposure that may influence fetal cardiovascular programming. Pregnancies complicated by preeclampsia and FGR are predominantly characterized by placental insufficiency and increased placental vascular resistance, resulting in chronic fetal pressure overload and altered afterload conditions. These hemodynamic disturbances are consistently associated with reduced ventricular deformation, altered chamber geometry, and early signs of myocardial functional adaptation. In contrast, GDM primarily exposes the fetus to a hyperglycemic and hyperinsulinemic intrauterine environment, promoting myocardial hypertrophy, altered myocardial energetics, and subtle systolic and diastolic dysfunction through metabolic rather than pressure-mediated mechanisms.

Despite these mechanistic differences, a convergent phenotype of subclinical myocardial dysfunction emerges across all three conditions, suggesting that distinct intrauterine stressors may ultimately activate shared fetal cardiac remodelling pathways. The degree, timing, and pattern of deformation abnormalities appear to be influenced by the relative contribution of placental dysfunction, hemodynamic load, and metabolic exposure, which may explain the heterogeneity observed across studies. This mechanistic framework highlights the importance of gestational timing and disease phenotype when interpreting 2D-STE findings and underscores the potential role of fetal myocardial deformation as an early marker of cardiovascular programming.

These divergent trajectories challenge a uniform “fetal programming” paradigm and indicate that the type, severity, and duration of intrauterine stress determine the onset and nature of cardiac adaptation. The relative preservation of function at mid-gestation identifies a potential therapeutic window before irreversible remodelling occurs, particularly in PE and GDM.

Methodological standardization remains the major barrier to clinical translation. Inter-vendor differences in strain algorithms, lack of gestational age-specific Z-scores, and variable definitions of FGR continue to hamper comparability. Until international consensus guidelines are implemented, routine mid-gestation STE cannot be recommended for risk stratification (Class III evidence).

Future research should prioritize large, prospective longitudinal cohorts integrating mid-gestation fetal STE with established placental biomarkers, such as placental growth factor, sFlt-1, and uterine artery Doppler indices, to better characterize the interaction between placental dysfunction and fetal myocardial adaptation. Serial fetal assessments across gestation, combined with standardized deformation imaging protocols, are needed to define temporal trajectories of cardiac remodelling rather than isolated cross-sectional differences. Importantly, long-term follow-up extending from the neonatal period into childhood and adulthood will be essential to determine whether early fetal deformation abnormalities translate into persistent cardiovascular phenotypes, including altered ventricular geometry, blood pressure regulation, or subclinical myocardial dysfunction. Linking fetal cardiac strain patterns to postnatal cardiometabolic outcomes may ultimately clarify the role of fetal myocardial phenotyping as a marker of developmental cardiovascular programming and inform future risk stratification strategies.

In conclusion, current evidence indicates that fetal myocardial function at mid-gestation remains largely preserved in pregnancies that subsequently develop PE, GDM or FGR despite the presence of early placental or metabolic stress. STE at 18–28 weeks, therefore, provides mechanistic insight into early cardiovascular adaptation rather than serving as a standalone predictive or diagnostic tool. Distinct temporal and pathophysiological trajectories appear to characterize each condition, with placental insufficiency primarily driving pressure-mediated remodelling in PE and FGR, and metabolic exposure contributing to later myocardial changes in GDM. Although subtle deformation abnormalities and mechanical dyssynchrony may represent early signatures of fetal cardiovascular programming, methodological heterogeneity and limited outcome correlation currently preclude routine clinical implementation. Future large-scale longitudinal studies integrating standardized fetal deformation imaging with placental biomarkers and long-term cardiovascular outcomes are essential to determine whether mid-gestation cardiac phenotyping can meaningfully refine risk stratification and personalized surveillance strategies in high-risk pregnancies.

## 5. Limitations

A very limited number of studies. Despite an exhaustive search up to November 2025, only 6 original datasets contributed mid-gestation STE data. This reflects the fact that routine application of fetal 2D-STE before 28 weeks remains confined to a handful of specialized tertiary centres.

Dominance of a single research group. Four of the 6 included datasets originate from the same fetal medicine unit at King’s College Hospital/St Thomas’ Hospital, London. This introduces a non-negligible risk of institutional bias in acquisition protocols, post-processing preferences, and interpretation of subtle strain differences.

Software and methodological heterogeneity. Three different commercial platforms (FetalHQ, TomTec, WMT) and one deep-learning automated system were used, with no universal Z-score normalization or consensus on region-of-interest definition. Absolute GLS values, therefore, vary by up to 4–5% between vendors for the same gestational age, making direct quantitative synthesis impossible and potentially masking or exaggerating true biological differences.

Paucity of truly pre-diagnostic prospective data. Only two large cohorts performed blinded STE at 19–24 weeks in unselected populations before any clinical or biochemical suspicion of the complication. The remaining studies are either smaller, high-risk selected, or retrospective in outcome assignment, reducing certainty that observed changes are genuinely predictive rather than early subclinical manifestations already present in high-risk groups.

Near-absence of early-onset severe FGR at mid-gestation. Mid-gestation STE data for severe, early-onset FGR are essentially limited to case reports and very small series (*n* < 10). The vast majority of available evidence, therefore, reflects late-onset FGR or constitutionally small fetuses, which have markedly different pathophysiology and cardiac trajectories.

Dyssynchrony-related limitations of GLS. Several included studies report mechanical dyssynchrony alongside altered GLS. Because GLS is computed by speckle-tracking algorithms that implicitly assume relatively synchronous myocardial deformation, GLS values may be less reliable in the presence of increased timing dispersion and may underestimate (or otherwise distort) true myocardial contractile performance. Consequently, comparisons of GLS between groups with differing degrees of dyssynchrony should be interpreted with caution, and GLS should ideally be contextualized with timing-based indices and conventional functional markers until further fetal-specific validation and standardization are available.

Outcome correlation. Lack of consistent linkage to perinatal outcomes and long-term follow-up; recommend explicit statement and a GRADE summary of certainty (low–moderate for most mid-gestation effects).

In addition to the other limitations discussed above, several study-level constraints warrant emphasis. Fetal heart rate was not standardized across the included studies, which may influence speckle-tracking–derived functional indices and contribute to between-study variability. Reproducibility was incompletely reported in multiple datasets, limiting the strength of conclusions regarding measurement reliability across centres and software pipelines. The available evidence also showed limited geographic and ethnic diversity, which may restrict generalizability and the transferability of findings to broader populations. Finally, some cohorts relied on birthweight-defined small-for-gestational-age as a surrogate for impaired growth; in these settings, true placental fetal growth restriction could not be confirmed antenatally, raising the possibility of phenotype misclassification and potentially diluting associations specific to placental insufficiency.

These limitations are inherent to an emerging field still in its infancy but must be acknowledged when interpreting the current evidence and, especially, when considering clinical implementation. They also define the precise roadmap for future research needed to move fetal mid-gestation STE from a research tool to a clinically actionable biomarker

## 6. Conclusions

2D-STE has substantially advanced the understanding of how maternal-placental disorders shape fetal cardiac development. Across conditions, mid-gestational alterations are subtle and adaptive, reflecting compensation rather than failure.

In PE, early changes are minimal, with mild LV alterations preceding later remodelling. In GDM, fetal function is preserved at 18–28 weeks and declines only after prolonged metabolic exposure. In FGR, early dyssynchrony signals adaptive strain redistribution that may progress to diastolic dysfunction as placental insufficiency worsens.

These findings are consistent with the hypothesis of fetal cardiovascular programming, whereby intrauterine stressors imprint long-term cardiac structure and function. While STE remains primarily a research tool, its integration into standardized fetal surveillance could enhance risk stratification and refine the timing of intervention in high-risk pregnancies. Future multicenter, longitudinal studies with standardized methodology and outcome validation will determine whether fetal STE transitions from a sophisticated research technique to a clinically actionable component of prenatal cardiac assessment.

## Figures and Tables

**Table 1:** Characteristics and main findings of studies performing fetal 2D-STE at mid-gestation in pregnancies that subsequently developed Preeclampsia, Gestational Diabetes or Fetal growth restriction [8,11,12,21,22,23].

First Author Year	Country	Design	Total	Complication	GA (wks)	Software	LV GLS	RV GLS	Other key STE	Main Conclusion	NOS
**Huluta 2023 [8]**	UK	Prospective cohort	5801	179 PE 149 GH	19–24 wks	WMT (Canon)	−22.2 (−23.3–−21.1) PE vs. −23.3 (−24.7–−21.9) Controls +0.8%	No diff.	LV EF ↓ 1.2%	Less negative GLS; clinically negligible.	8
**Huluta 2023 [11]**	UK	Prospective cohort	5620	470 GDM	19–24	WMT (Canon)	No diff.	No diff.	septal thickness ↑ left atrial area ↑ LV EF ↑ 0.02%	No functional change before diagnosis.	8
**Yovera 2021 [12]**	Multicenter	Cohort comparison	336	112 GDM	24–40	TomTec	No difference	Reduced	RV TAPSE ↓	In GDM, reduced RV systolic function.	6
**Wang 2021 [21]**	China	Prospective cohort	116	58 GDM	24–40	Fetal HQ	−18.26 ± 1.39 GDM vs. −22.70 ± 1.34 Controls	−18.52 ± 1.35 GDM vs. −22.74 ± 1.34 Controls	-	Biventricular systolic dysfunction and a rounder cardiac shape in GDM fetuses from 24 weeks onward (post-diagnosis)	7
**Neacșu 2025 [22]**	Romania	Case study	2	2 severe early-FGR	20–22	TomTec	Case 1: −15.2 Case 2: −14.8	Case 1: −16.5%; Case 2: −15.9%	-	In severe early-onset FGR, reduced biventricular GLS and increased dyssynchrony detectable at 20–22 weeks before overt Doppler changes.	5
**van Oostrum 2020 [24]**	Netherlands	Prospective	136	12 Future FGR	18–23	TomTec	No diff. until > 26 wks	−20.24 (−16.29 to − 24.28) in controls vs. −15.98 (−11.69 to −20.55) in future FGR		RV systolic dysfunction (less negative GLS) is detectable from 18 to 21 weeks in fetuses who later become FGR—earliest functional marker reported.	8

Values are presented as reported by authors (mean ± SD or median [IQR]); negative GLS values indicate a greater magnitude of deformation. Less negative GLS (impaired systolic function). Abbreviations: GA = gestational age at scan; PE = preeclampsia; GH = gestational hypertension; GDM = gestational diabetes mellitus; FGR = fetal growth restriction; STE = speckle tracking echocardiography; GLS = global longitudinal strain; EF = ejection fraction; RV = right ventricle; LV = left ventricle; NOS = Newcastle–Ottawa Scale score (0–9); wks = weeks.

## Supplementary Materials

The following supporting information can be downloaded at https://www.mdpi.com/article/10.3390/jcm15051845/s1, Table S1. Detailed literature search strategy.
