# Supplementary material for: Alteration in Fetal Cardiac Function at Mid-Gestation Among Pregnancies Subsequently Complicated by Preeclampsia, Fetal Growth Restriction and Gestational Diabetes Mellitus: A Literature Review
Source: J Clin Med. 2026 Feb 28;15(5):1845. doi: 10.3390/jcm15051845 (PMC12985935; doi:10.3390/jcm15051845)
Supplement: Supplementary file 1 [file jcm-15-01845-s001.zip › jcm-4085400-supplementary.docx]

| Database | Search Strategy | Date Searched | Filters |
| --- | --- | --- | --- |
| PubMed | ("Speckle Tracking Echocardiography"[MeSH] OR speckle tracking OR strain imaging OR myocardial deformation) AND (fetal OR fetus OR prenatal) AND (preeclampsia OR gestational diabetes OR fetal growth restriction) | 1 Nov  2025 | English, Humans |
| Embase | (speckle tracking OR strain imaging OR fetal ecocardiography) AND (fetus) AND (preeclampsia OR GDM OR FGR) | 1 Nov  2025 | English |
| Scopus | TITLE-ABS-KEY ("speckle tracking" OR "strain imaging" OR "myocardial deformation" OR "global longitudinal strain" OR GLS) AND (fetal OR fetus OR prenatal OR antenatal) AND (preeclampsia OR "hypertensive disorders of pregnancy" OR "fetal growth restriction" OR FGR OR IUGR OR "gestational diabetes" OR GDM) AND (echocardiography OR ultrasound) | 1 Nov  2025 | English, Human studies |

Table S1. Detailed literature search strategy
